# Weight, Blood Pressure, and Dietary Benefits After 12 Months of a Web-based Nutrition Education Program (DASH for Health): Longitudinal Observational Study

**DOI:** 10.2196/jmir.1114

**Published:** 2008-12-12

**Authors:** Thomas J Moore, Nour Alsabeeh, Caroline M Apovian, Megan C Murphy, Gerald A Coffman, Diana Cullum-Dugan, Mark Jenkins, Howard Cabral

**Affiliations:** ^2^Boston University School of Public HealthBostonMAUSA; ^1^Boston University School of MedicineBostonMAUSA

**Keywords:** Weight loss, blood pressure, hypertension, health education, diet, Internet, behavior change

## Abstract

**Background:**

The dietary habits of Americans are creating serious health concerns, including obesity, hypertension, diabetes, cardiovascular disease, and even some types of cancer. While considerable attention has been focused on calorie reduction and weight loss, approaches are needed that will not only help the population reduce calorie intake but also consume the type of healthy, well-balanced diet that would prevent this array of medical complications.

**Objective:**

To design an Internet-based nutrition education program and to explore its effect on weight, blood pressure, and eating habits after 12 months of participation.

**Methods:**

We designed the DASH for Health program to provide weekly articles about healthy nutrition via the Internet. Dietary advice was based on the DASH diet (Dietary Approaches to Stop Hypertension). The program was offered as a free benefit to the employees of EMC Corporation, and 2834 employees and spouses enrolled. Enrollees voluntarily entered information about themselves on the website (food intake), and we used these self-entered data to determine if the program had any effect. Analyses were based upon the change in weight, blood pressure, and food intake between the baseline period (before the DASH program began) and the 12th month. To be included in an outcome, a subject had to have provided both a baseline and 12th-month entry.

**Results:**

After 12 months, 735 of 2834 original enrollees (26%) were still actively using the program. For subjects who were overweight/obese (body mass index > 25; n = 151), weight change at 12 months was -4.2 lbs (95% CI: -2.2, -6.2; *P* < .001). For subjects with hypertension or prehypertension at baseline (n = 62), systolic blood pressure fell 6.8 mmHg at 12 months (CI: -2.6, -11.0; *P* < .001; n = 62). Diastolic pressure fell 2.1 mmHg (*P* = .16). Based upon self-entered food surveys, enrollees (n = 181) at 12 months were eating significantly more fruits, more vegetables, and fewer grain products. They also reduced consumption of carbonated beverages. Enrollees who had visited the website more often tended to have greater blood pressure and weight loss effect, suggesting that use of the DASH for Health program was at least partially responsible for the benefits we observed.

**Conclusions:**

We have found that continued use of a nutrition education program delivered totally via the Internet, with no person-to-person contact with health professionals, is associated with significant weight loss, blood pressure lowering, and dietary improvements after 12 months. Effective programs like DASH for Health, delivered via the Internet, can provide benefit to large numbers of subjects at low cost and may help address the nutritional public health crisis.

## Introduction

The dietary habits of Americans are creating serious health concerns. The “obesity epidemic” is the most publicized evidence of the problem, but it is only one aspect. Studies have suggested that better dietary habits can, even with only modest weight loss, prevent or help control a number of expensive, chronic conditions like hypertension, cardiovascular disease, diabetes, and even some types of cancer [1-5].

There is a growing need for effective ways to improve Americans’ eating habits, but it is difficult to change dietary habits and maintain those changes. Weight loss studies have shown short-term success but gradual regain of weight in the longer term [6]. New approaches are needed that can achieve long-term success at low cost. One promising approach is the use of the Internet. Web-based programs can be developed and delivered to large segments of the population relatively inexpensively. There is some evidence that use of these programs can lead to short-term weight loss [7], but there is little evidence that they are effective “wellness” programs, achieving not just weight loss but other health benefits as well.

We designed a Web-based program, DASH for Health, to improve nutrition and physical activity habits. The nutrition advice was based on the DASH Diet (Dietary Approaches to Stop Hypertension) [1]. Although the DASH Diet was originally developed to prevent or treat high blood pressure, it is essentially a well-balanced diet that is now recommended by the USDA Dietary Guidelines for Americans, 2005 as being an ideal eating pattern for all American adults [8]. The DASH Diet also has the support of the NHLBI. The DASH for Health program was developed to improve eating habits in the general population, not just to treat overweight and obesity. The goal of this study was to explore the effects of the DASH for Health program over the course of one year in 2834 enrollees.

## Methods

### Research Subjects

The DASH for Health program was offered as a free employee benefit to all US-based employees (approximately 12,500) of EMC Corporation, a global information infrastructure company based in Hopkinton, Massachusetts. The program was also offered to all adult household members of these employees. Employees and household members were encouraged to join the online program through a series of email communications from EMC leaders. During a three-week open enrollment period, 3479 subjects enrolled in the program and logged on to the website at least once. At the time of enrollment, we asked enrollees if we could use information that they entered about themselves on the website (eg, weight, blood pressure (BP) levels, food intake) to determine whether the program was providing benefit. This report is based upon the 2834 enrollees (81%) who granted consent.

The project was approved by the Institutional Review Board of Boston University Medical Center.

### The DASH for Health Program

**Figure 1:**
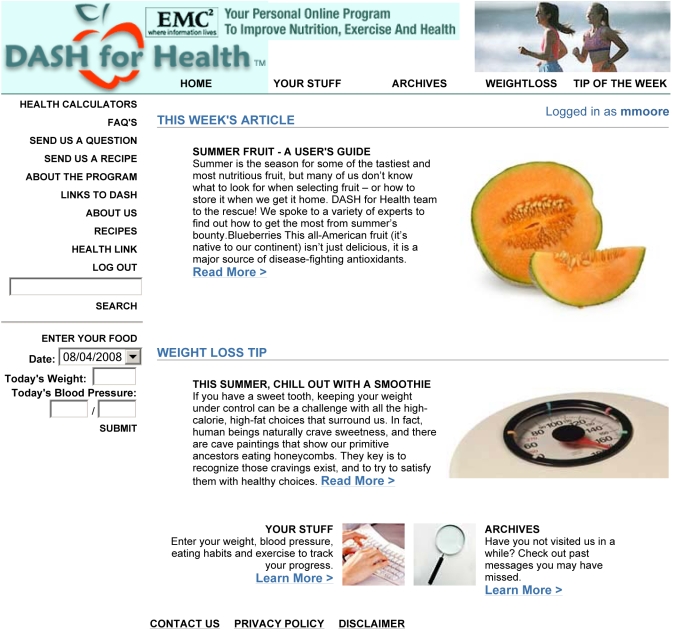
A view of the program’s homepage as designed for EMC Corporation employees

Enrollees were given access to a personalized, password-protected website. Figure 1 displays a view of the program’s homepage as designed for the EMC audience. Through that website, we published new articles once a week. The articles contained information about elements of healthy nutrition. About 15% of the articles also dealt with healthy exercise practices. A sub-article each week addressed specific issues to promote weight loss. Articles were typically published every Friday and a reminder email was sent to each enrollee at the time a new article was posted on the website. The emails contained a brief description of that week’s article and a hyperlink to the log-in website. Nutrition advice was based on the DASH Diet, meaning that we instructed people about consuming various servings of the eight DASH food groups (fruits, vegetables, low-fat dairy, meat/fish/poultry, grains, nuts/legumes, sweets, and added fats). The articles were not targeted to specific subsets of the enrollee population. All enrollees received the same articles.

Based on an enrollee’s gender, age, and activity pattern, algorithms on the website calculated the number of servings of each DASH food group the enrollee should consume each day. Enrollees were encouraged to enter information about themselves on the website such as weight, blood pressure, and 24-hour food recall using a recall instrument which converted common foods into servings of DASH food groups. This DASH recall instrument was designed for this program and was validated against the Block 98.2 Food Frequency Questionnaire (data not shown). The website converted those self-entered data into progress report graphs. Although enrollees had the option of submitting email questions for the investigators to respond to, we designed the program to provide minimal personal contact. The goal was to develop a program which, with only minimal person-to-person interaction, could influence behaviors.

We did not impose any limits or expectations on how enrollees used the website. They were free to select for themselves which articles to read and how frequently to enter information about their weight, blood pressure, or eating habits.

### Outcome Measures

We had three primary outcomes, all measured at 12 months: first, change in weight between baseline and 12 months in subjects who indicated a desire to lose weight on their enrollment questionnaire; second, change in systolic blood pressure (SBP) in those who indicated that they either had high blood pressure or were on blood pressure medications or had been told to watch blood pressure (we defined this as our “High Blood Pressure” group); and third, change in consumption of DASH food groups. Change in diastolic blood pressure (DBP) was a secondary outcome. We also performed exploratory analyses of the relationship between our outcomes and the amount of use of the DASH for Health website.

For weight measurement, we used self-entered weights from the website which may have included weights taken by the subjects themselves or taken in other settings (eg, physician visits). We classified subjects as overweight/obese based on their body mass index (BMI; kg/m^2^). Similarly for blood pressure, we used self-entered readings which could have been self-measurements or readings taken by others. We did provide recommendations on the website about how to take one’s own blood pressure (seated, left arm, average of two measurements). In addition, the employer, EMC Corp., offered free automated sphygmomanometers (Fore-Care 6400; Forecare Inc., Buffalo Grove, IL) to enrollees with hypertension. Food consumption was estimated from the DASH Online Questionnaire, a 24-hour recall instrument. For weight, blood pressure, and food intake, if there were more than a single entry during the baseline or 12th-month time window, we averaged the entries and used that single value in our analyses. Website use was calculated as the number of log-ons by each enrollee who visited the website (unique users).

### Data Analysis

For our analyses, we used the data that enrollees self-entered on the website. There was no randomization and no control group. Our analyses do not allow estimation of what the effects of DASH for Health might have been on enrollees who did not enter any data. The baseline data reflect information that enrollees entered on the DASH for Health website during the three-week enrollment period (before the website was delivering any behavior-changing messages), and the “12th-month” data are those entered during weeks 48-52. The number of subjects analyzed for each outcome was determined by the number of subjects who entered data for that outcome during both the baseline and 12th-month time frame. In analyzing website use, we used the number of log-ins over 12 months. Data are displayed as means unless otherwise noted, and indices of dispersion are standard deviation (SD) or 95% confidence intervals (CI) as noted. All analyses were performed with SigmaStat 3.5. Baseline versus 12th-month comparisons were performed with paired *t*-tests or Wilcoxon signed rank tests. Statistically significant results had *P* values less than .05.

## Results

### Subjects

Enrollees were widely distributed geographically, residing in 41 states. They were approximately evenly distributed by gender, and their ages ranged from 18-73 years (average 40.7 years). They were highly educated, with 1845 (66%) having completed college or postgraduate work. Of the subjects, 88% were white, and 74% were married (see Table 1 for absolute numbers of subjects). The most commonly stated reasons for enrolling in DASH for Health were desires for general health information and weight loss. Approximately 25% were also concerned about blood pressure or cholesterol levels. Other demographic details are shown in Table 1.

Of the 3479 subjects who enrolled in the program and logged on to the website at least once, 2834 (81%) granted consent to use their data for research purposes. Of these, 735 (26%) were still actively using the website in the 12th month. Their demographics are also shown in Table 1. The groups were comparable except that subjects in the 12-month group were older, included a greater percentage of women, had fewer single and more widowed subjects, and had a greater percentage who were interested in “general health information” at the time of enrollment.

**Table 1:** Characteristics of all enrollees at baseline and of those still using the program at 12 months, using self-entered data at time of enrollment

		All Enrollees n (%)	Still Active at 12 Months n (%)	*P* Values
All enrollees		2834	735	
Males		1568 (55%)	369 (50%)	.01
Females		1266 (45%)	366 (50%)	.01
Average Age (years)		40.7	42.2	.001
Average Weight (lbs)		182.7	179.8	.11
**Education-lowest level achieved**				
	Grade School	34 (1%)	5 (< 1% )	.23
	Some High School	10 (< 1%)	1 (< 1%)	.34
	Completed High School	171 (6%)	35 (5%)	.19
	Some College	733 (26%)	175 (24%)	.25
	Completed College	1140 (41%)	307 (43%)	.45
	Postgraduate Work	705 (25%)	199 (28%)	.22
**Marital Status**				
	Single	522 (19%)	110 (15%)	.03
	Widowed	19 (1%)	11 (2%)	.03
	Married	2063 (74%)	548 (76%)	.34
	Divorced/Separated	190 (7%)	52 (7%)	.72
**Ethnic Status**				
	African American	62 (2%)	13 (2%)	.48
	Native Hawaiian/Pacific Islander	20 (1%)	4 (1%)	.63
	White	2470 (88%)	648 (90%)	.46
	American Indian	7 (< 1%)	1 (< 1%)	.57
	Native American	13 (< 1%)	4 (1%)	.76
	Hispanic	67 (3%)	17 (3%)	.93
	Other	221 (8%)	51 (7%)	.43
**Reasons for Enrolling and Health Concerns** (from enrollment questionnaire)				
	Want general health info	2204 (78%)	604 (82%)	.01
	Weight concern^a^	2160 (76%)	568 (77%)	.54
	High Blood Pressure^b^	664 (24%)	195 (27%)	.08
	Have diabetes	98 (3%)	21 (3%)	.42
	Have high cholesterol	790 (28%)	206 (28%)	.93

^a^“Weight concern” group includes subjects who indicated they wanted to lose weight.

^b^“High Blood Pressure” group includes subjects who indicated one or more of the following: have high blood pressure; are taking antihypertensive medications; have been told by doctors to “watch” their blood pressure.

### Website Use

**Figure 2:**
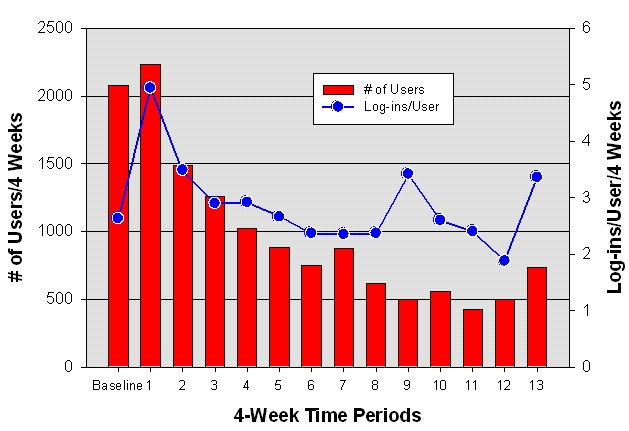
Pattern of website use during a 3-week baseline period and then in sequential 4-week periods for 12 months

At the end of 12 months, 735 of the original 2834 enrollees (26%) were still actively visiting the website. Figure 2 displays the pattern of website use during a 3-week baseline period and then in sequential 4-week periods for 12 months. The dropout rate was highest during the first 2 months. The number of users stabilized and remained fairly constant for the final 5 months. On average, users visited the website two to three times during any 4-week period. However, it was not the same group of users who visited the website during each 4-week period: enrollees were dropping out and dropping back in over the entire 12 months.

### Weight Change

There were 203 subjects who indicated a desire to lose weight when they enrolled in the program and who entered their weight during the baseline period and during the 12th month of the program. Their average weight change was -3.1 lbs (CI -4.7, -1.5; *P* < .001). The overall range of weight change was +30 to -62 lbs. Of these 203 subjects, 151 had BMI in the overweight/obese range, ranging from 25.0 to 50.6 kg/m^2^. This was the subgroup for our weight-loss primary outcome. Their average weight change was -4.2 lbs (CI -6.2, -2.2; *P* < .001). Of these, the 74 obese subjects (BMI ≥ 30) lost 5.2 pounds while the 77 overweight subjects (BMI ≥ 25 to 29.9) lost 3.4 pounds (weight change in obese versus overweight, *P* = .63). For the remaining 53 subjects, with BMI 18.5 - 24.9 kg/m^2^, the average weight change was +0.2 lbs. Other characteristics of these subjects are shown in Table 2.

**Table 2:** Weight change from baseline to 12 months

	Baseline weight mean lbs (SD)	Weight change mean lbs (95% CI)	Age yrs (SD)	Males n (%)	Females n (%)
All subjects (n = 203)	187.0 (43.0)	-3.1 (-1.5, -4.7)^a^	42.7 (10.0)	79 (39)	124 (61)
BMI > 25 (n = 151)	202.3 (38.3)	-4.2 (-2.2, -6.2)^a^	43.2 (9.9)	72 (48)	79 (52)
BMI < 25 (n = 52)	142.4 (16.7)	+0.2 (-1.6, +2.0)	41.6 (10.0)	7 (13)	45 (87)

^a^Weight change in *all* subjects and BMI > 25 groups: *P* < .001.

### Blood Pressure Change

**Figure 3:**
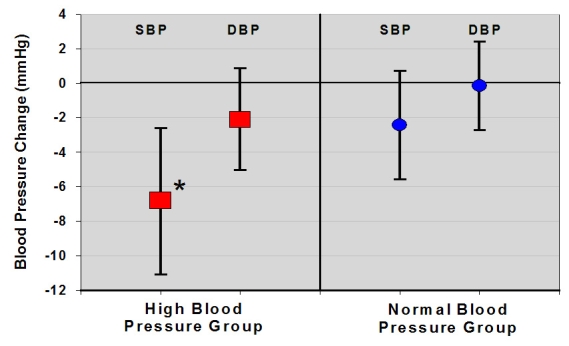
Mean (+ /- 95% CI) systolic and diastolic blood pressure change at 12 months in high and normal blood pressure groups (the systolic change in the high blood pressure group was significant, *P* < .001)

A total of 120 subjects entered blood pressure readings on the website during the baseline period and the 12th month (Table 3 and Figure 3). Of these, 62 met the definition of our high blood pressure group. Their systolic pressure change was -6.8 mmHg (*P* < .001); diastolic change was -2.1 mmHg (*P* = .16). An additional 58 subjects who indicated no blood pressure concern on their baseline questionnaires also entered blood pressure recordings in the baseline and 12th month. Their baseline blood pressure was lower than in the high blood pressure group (Table 3), and their systolic and diastolic pressure change was -2.4/-0.2 mmHg (*P* = .09 and .90, respectively).

**Table 3:** Blood pressure change

	Baseline BP mmHg	Systolic change mean (95% CI)	Diastolic change mean (95% CI)	Age yrs (SD)	Males n (%)	Females n (%)
High Blood Pressure group (n = 62)	137.3/81.2	-6.8 (-2.6, -11.0)^a^	-2.1 (+0.8, -5.0)	48.6 (7.7)	32 (52)	30 (48)
No High Blood Pressure (n = 58)	118.0/73.5	-2.4 (+1.3, -6.1)	-0.2 (+2.4, -2.8)	41.0 (9.1)	22 (38)	36 (62)

^a^Systolic change in high blood pressure group: *P* < .001

### Change in Dietary Habits

A total of 181 enrollees completed at least one DASH online food questionnaire during the baseline period and the 12th month. The median number of completed questionnaires per enrollee was three during baseline and three during the 12th month. The average age was 42.4 years; 107 were women; 74 were men. Table 4 displays the average DASH goal (as servings / day) for each of the eight DASH food groups, as well as the number of servings consumed (as entered in the DASH online questionnaire). Consistent with DASH recommendations, there were significant increases in daily fruit and vegetable intake in the group. There was also a significant decrease in the consumption of grain products, moving counter to the DASH goal. There were no significant changes in any of the other five DASH food groups.

The DASH online questionnaire also provided information on 52 subcategories of these eight main food groups. We performed exploratory analyses to examine changes in these subcategories. There were several significant changes in subgroup consumption. Three categories merit mention. Consumption of carbonated beverages decreased from 9 oz per day to 6.5 oz (*P* < .001). Enrollees reported reducing their consumption of refined-wheat bread products by 0.4 servings per day while increasing consumption of whole grain bread products by 0.3 servings per day (*P* < .001 and *P* = .004, respectively). So, while grain consumption overall decreased, the shift from refined grains to whole grains is consistent with DASH advice.

**Table 4:** Changes in consumption of the eight main DASH food groups from baseline to the 12th month of DASH for Health (n = 181)

	DASH Goals (servings/d)	Average Baseline (servings/d)	Average 12th month (servings/d)	Difference (12th month minus baseline)	*P*
Fruit	4	2.0	2.2	+0.2	.03
Vegetables	4	2.6	3.1	+0.5	.002
Grains	7	4.4	4.2	-0.2	.04
Dairy	2.5	2.1	2.0	-0.1	.48
Meat/fish	1.5	1.9	1.9	0	.30
Nuts/beans	0.5	0.4	0.5	+0.1	.76
Added fats	2	1.6	1.5	-0.1	.15
Sweets	0.5	1.3	1.2	-0.1	.13

### Relationship Between Website Use and Outcomes

We performed exploratory analyses, relating the amount of website use (measured as number of log-ins over the course of 12 months) versus change in our main outcomes: weight, systolic blood pressure, and consumption of DASH food groups. We divided the sample into two parts based on the median number of log-ins. The median log-in number differed for each outcome, being determined by the number who provided baseline and 12-month data for that outcome. For weight and blood pressure, there were tendencies toward greater effect among those with more log-ins (Table 5). The only significant difference in food group consumption (data not shown) was greater fruit intake among those with a greater number of log-ins (*P* = .03). There were no differences in other food groups. The median log-in number for the group who provided dietary data (food questionnaires) was 44.

**Table 5:** Comparison of changes in weight and blood pressure in relation to number of DASH website log-ins (median log-ins for blood pressure group was 50; median for weight group was 40)

	≤ Median log-ins	> Median log-ins	*P* (≤ median vs > median)
Systolic BP (mmHg; CI)	-3.9 (-9.9, +2.2)	-9.8 (-15.9, -3.7)	.06
Diastolic BP (mmHg; CI)	+0.7 (-3.8, +5.1)	-4.8 (-8.6, -1.0)	.06
Weight (pounds; CI)	-1.5 (-3.5, +0.5)	-4.6 (-6.9, -2.3)	.09

## Discussion

We have found that an online program that provides weekly educational information, motivational messages, and convenient ways for self-monitoring can lead not just to significant weight loss but also to reduction in blood pressure and to healthier dietary habits.

Our results compare favorably to other programs. In terms of retention in the program, 735 of the original 2834 enrollees (26%) were still using the DASH for Health website after 12 months. Very little has been published about long-term subject retention in lifestyle improvement programs in real-world settings, but, as one example, Finley et al reported that, of > 60,000 enrollees in the Jenny Craig program (not Internet-based), only 6.6% were still retained in the program at 52 weeks [9]. One important difference between that program and ours that may have affected retention is that it is expensive (> $1000/year) compared to ours which was free to enrollees. The overweight/obese enrollees in our program lost 4.2 pounds after one year, meeting the definition of an “effective” program as defined in the Center for Disease Control’s review of worksite strategies for weight control [8]. The 6.8 mmHg reduction in systolic pressure represented 60% of the systolic change seen in the hypertensive subjects in the original DASH trial, which was a controlled feeding study [1]. The fact that the weight and blood pressure changes in our study tended to be greater in those enrollees who used the website more often suggests that use of the DASH for Health program was at least partially responsible for these improvements.

There are thousands of websites on the Internet that provide nutrition information, including more than 400,000 websites that mention the DASH diet. Most of these websites provide static content and are not true education programs. Those that are actual education programs, such as eDiets.com or Weight Watchers, focus on weight loss, and there is little evidence that they provide long-term benefit. Womble et al assessed the weight loss effect of eDiets.com for 12 months in 23 women [10]. The women lost on average 0.8 kg. In addition to the standard, online eDiets program, subjects also had five one-on-one sessions with a psychologist which may have enhanced eDiets’ effect, since, in other studies, person-to-person intervention seems to increase the results of an online program (see below).

The Internet has also been used in other ways in research studies. Some investigators have used it as a communication tool between an individual nutritionist and a client (a strategy to extend a nutritionist’s reach to greater numbers of clients). In general, programs with more intense or frequent person-to-person interaction lead to greater retention and health benefits [11-18]. As an example, Tate et al conducted a 6-month randomized controlled trial comparing the weight loss effects of an online program with various levels of additional support: no additional support versus computer-generated feedback versus personalized email from a nutrition counselor [12]. Weight loss at 6 months was related to type of interaction, ranging from -2.6 kg in the no feedback group to -7.3 kg in the personal email group, yet even the no-feedback group in this study had a one-hour counseling session at baseline as well as orientation on how to use the website. Subjects were also given free meal replacements (Slim-Fast) for two meals per day for one week and then coupons for discounted meal replacements for the remainder of the study. In these trials of online approaches to weight loss, the person-to-person intensity of even the least intense treatment arm was greater than what was provided in the DASH for Health program, which we deliberately designed to minimize interactions between the participants and the program team in an attempt to reduce the cost of operating the program. The results of these former studies suggest that adding personal email contact or adding face-to-face sessions may modestly increase the amount of weight loss, although such additions would have also dramatically decreased the scalability of our program and increased its cost.

We believe that scalability and cost are important considerations when addressing a problem as vast as the eating habits of the roughly 140,000,000 Americans who have nutritionally-related health concerns. The physicians and nurses who form the framework of our health care system do not have the time or, in many cases, the background training to counsel patients about nutrition. Additionally, most health insurance products limit the number of allowable visits with a nutritionist. An approach is needed that can be offered without imposing additional burdens on our health care workers or on our health care budget. The Internet, in our view, could potentially provide such a solution.

There were some limitations to this study. First, we relied totally on self-entered data, with no objective measurements to confirm the self-entered results. Second, we could only assess changes in our outcomes after 12 months in people who were, by definition, still using the website. Even though there were no demographic differences between the 12-month users versus all those who enrolled at baseline, it is likely that this group was highly self-selected: people who continued to use the website for the entire year probably did so, in part, because they were deriving some benefit from the website (as observed by Finley et al [9]), although not everyone who reported a weight or blood pressure change after 12 months showed benefit. Relying on self-entered data after 12 months, however, could introduce a bias toward positive benefits for our program. On the other hand, the fact that we minimized contact with the enrollees, specifically avoiding individual contact, and did not take objective measurements of outcomes allowed us to assess how subjects would use a program like ours in a real-world setting. This could be considered an advantage. Another limitation is the absence of a control group: we cannot compare the findings in DASH enrollees against a group of non-enrollees. The one mitigating observation here is that subjects who used the DASH website less often tended to lose less weight and had less blood pressure reduction than those who used the website more often, suggesting that use of the DASH program contributed to the weight and blood pressure changes. Overall, however, our reliance on self-entered data, a self-selected group of 12-month users, and the lack of a control group must be seen as significant limitations to our findings.

In summary, we showed that 26% of original enrollees continued to use the Web-based DASH for Health program at the end of one year and that, at one year, those who continued using the program had not only lost weight but also lowered their blood pressure and made healthy changes in dietary habits. While this study does not prove a causal relationship between using the program and achieving healthy changes, the possibility that well-designed, Internet-based programs can produce or aid in achieving important health benefits is encouraging. Programs like this one could play an important part in our efforts to improve the way Americans eat.

## Abbreviations

BMI: body mass index
BP: blood pressure
DASH: dietary approaches to stop hypertension
DBP: diastolic blood pressure
NHLBI: National, Heart, Lung, and Blood Institute
SBP: systolic blood pressure
USDA: United States Department of Agriculture
